# Antibacterial/Antifungal Activity and Synergistic Interactions between Polyprenols and Other Lipids Isolated from *Ginkgo Biloba* L. Leaves

**DOI:** 10.3390/molecules18022166

**Published:** 2013-02-07

**Authors:** Ran Tao, Cheng-Zhang Wang, Zhen-Wu Kong

**Affiliations:** 1 Institute of Chemical Industry of Forest Products, CAF, Nanjing 210042, Jiangsu, China; 2 National Engineering Laboratory for Biomass Chemical Utilization, Nanjing 210042, Jiangsu, China; 3 Key and Open Laboratory on Forest Chemical Engineering, SFA, Nanjing 210042, Jiangsu, China; 4 Key Laboratory of Biomass Energy and Material, Nanjing 210042, Jiangsu, China; 5 Institute of New Technology of Forestry, CAF, Beijing 100091, China

**Keywords:** *Ginkgo biloba*, polyprenols, lipids, antibacterial activity, antifungal activity, synergistic effect

## Abstract

Polyprenols separated from lipids are promising new components from *Ginkgo biloba* L. leaves (GBL). In this paper, ginkgo lipids were isolated by extraction with petroleum ether, saponification, and molecular distillation. Eight known compounds: isophytol (**1**), nerolidol (**2**), linalool (**3**), *β*-sitosterol acetate (**4**), *β*-sitosterol (**5**), stigmasterol (**6**), ergosterol (**7**), *β*-sitosterol-3-*O*-*β*-D-glucopyranoside (**8**) and *Ginkgo biloba* polyprenols (GBP) were separated from GBL by chromatography and identified mainly by NMR. The separated and identified compounds **1**, **2** and **3** are reported here for the first time in GBL. The 3D-DAD-HPLC-chromatogram (190–232 nm) of GBP was recorded. This study provides new evidence as there are no previous reports on antibacterial/antifungal activities and synergistic interactions between GBP and the compounds separated from GBL lipids against *Salmonella enterica*, *Staphylocococus aureus* and *Aspergillus niger*. Nerolidol (**2**) showed the highest activity among all the tested samples and of all mixture groups tested the GBP with isophytol (**1**) mixture had the strongest synergistic effect against *Salmonella enterica* among the three tested strains. A proportion of isophytol and GBP of 38.19%:61.81% (wt/wt) was determined by mixture design as the optimal proportion for the synergistic effect of GBP with isophytol against *Salmonella enterica*.

## 1. Introduction

*Ginkgo biloba* L., the sole surviving species of the division Ginkgophyta, is considered as a living fossil due to its survival for over 180 million years. *Ginkgo biloba* L. leaves (GBL), as a Traditional Chinese Medicine, are commonly used in the clinic in China. This has also been reported in many famous Chinese herbal treatises, such as Shen Nong Ben Cao Jing (2,800 BC) and Pen Ts’ao Kang Mu (1596) [1]. GBL contain many kinds of bioactive components such as flavonoids, biflavones, proanthocyanidins, alkylphenols, carboxylic acids, sterols, polyprenols, and so on [2]. Ageta’s research concluded that ginkgo lipids mainly consisted of 10% of fatty acids, 15% of esters, 75% of wax esters, aldehydes and long-chain alkanols [3]. It was shown in the 1970s that the non-saponifiable fraction of GBL lipids could be crystallized from alcohol-acetone and ethyl acetate to yield *β*-sitosterol, [4]. Nguyen Tu *et al.* used GC/MS to analyze the chemical composition of *Ginkgo biloba* L. lipids from external and internal leaves and identified some compounds, including several series of phenolic constituents and chainlike alcohols (ketones) [5]. It was reported that non-saponifiable lipids of GBL contained terpenoids, polyprenols, sterols, chainlike alcohols (ketone, ester) and so on. Crystalline solids containing mainly sterols were isolated from the non-saponifiable fraction of GBL by saponification, extraction, refrigeration and recrystallization [6]. *Ginkgo biloba* polyprenols (GBP), new natural active components discovered after ginkgo flavonoids and terpene lactones, mainly exist in the form of acetates and are difficult to separate from other lipids. GBP is generally composed of 15 to 21 unsaturated isoprene units and is one type of betulaprenol with an *E*,*E*-farnesyl residue at the ω-end of the prenyl chain and terminated by an isoprene unit bearing a primary hydroxyl group [7]. Reviews on GBP and chromatography of GBP in general were published at 2000 [8]. Wang *et al.* researched the separation of GBP enriched in heavy GBL distillates by molecular short-path distillation [9]. We recently reported on analysis of light distillates containing mainly volatile oil, chainlike alcohols (ketones, esters) and sterols separated by molecular distillation from the non-saponifiable fraction of GBL based on Py-GC-MS [10]. However, there are still no systematic reports on the separation and purification of GBP and other co-existing lipids. On the basis of these studies, it is important to separate and identify main compounds of non-saponifiable lipids from GBL by chromatography and spectrometry for elucidating and further studying these non-saponifiable lipids of GBL.

**Figure 1:**
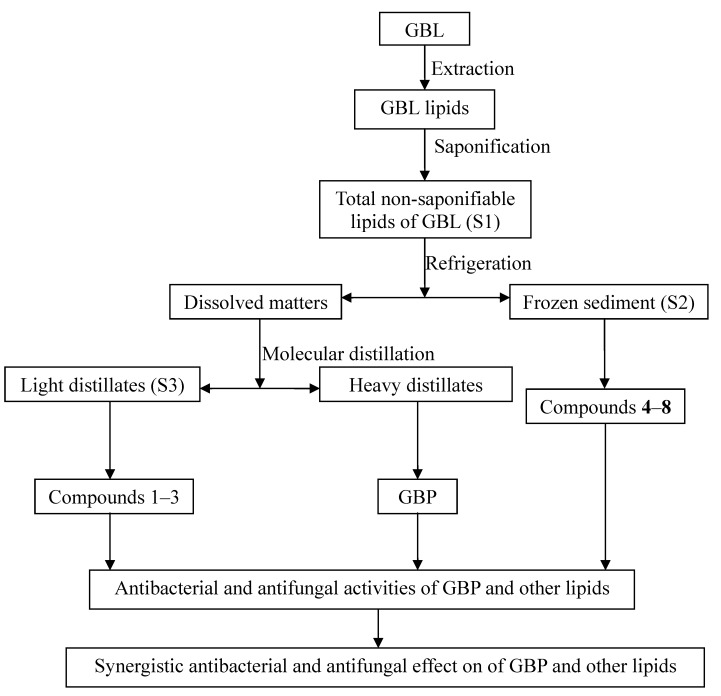
The study route of extraction, isolation and synergistic antibacterial/antifungal effects on GBP and other lipids from GBL.

Biological activities of *Ginkgo* extracts and constituents against bacteria, insects, and fungi were reported [11]. Sati *et al.* reported that hexane extracts of GBL showed actovoty against five pathogenic strains [12]. A chloroform fraction prepared from the sarcotesta of GBL where the active compounds were identified as salicylic acids showed potent inhibitory activity against vancomycin-resistant *Enterococcus* (VRE) [13]. Therefore we infer that some fat-soluble components of GBL have activity against some specific types of microorganisms. However, there are no evaluations on inhibition of microorganisms by GBL lipids, including GBP. Recently, we found that GBP and compounds separated from GBL lipids had antibacterial/antifungal activities and synergistic interactions *in vitro*. In this paper, we report that eight known compounds and GBP can be separated from non-saponifiable lipids of GBL by saponification, refrigeration, chromatography and identified by NMR analysis. The antibacterial/ antifungal activities and synergistic interactions between GBP and compounds separated from GBL lipids are examined according to the study route followed (Figure 1).

## 2. Results and Discussion

### 2.1. Structure Determination of Separated Compounds

**Figure 2:**
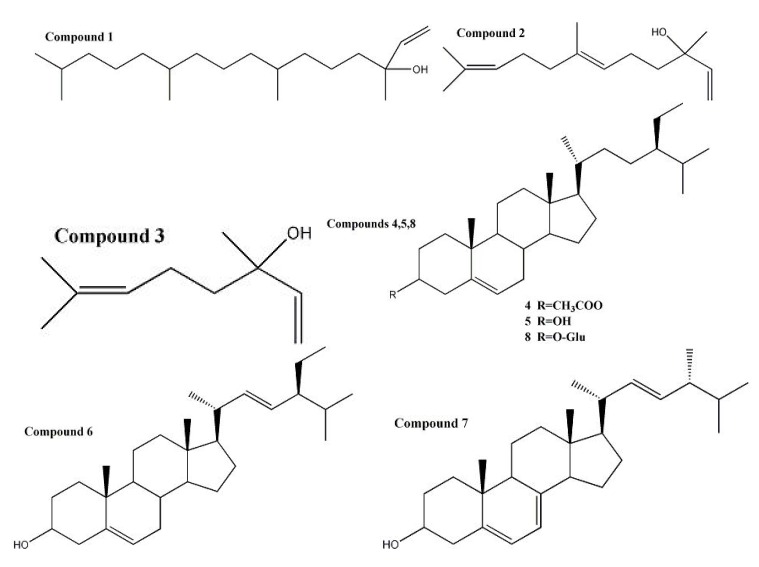
hemical structures of compounds **1**–**8** separated from GBL.

In total eight known compounds were separated from the different polar portion of the frozen sediment (**S2**) and the light distillates (**S3**) that were collected from the non-saponifiable lipids of GBL by chromatography and eight compounds were identified using spectroscopic techniques, mainly ^1^H- and ^13^C-NMR, as isophytol (**1**) [14], nerolidol (**2**) [15], linalool (**3**) [16], *β*-sitosterol acetate (**4**) [17], *β*-sitosterol (**5**) [18], stigmasterol (**6**) [19], ergosterol (**7**) [19] and *β*-sitosterol-3-*O*-*β*-D-glucopyranoside (**8**) [20] by comparison with the data of corresponding references (Figure 2).

**Figure 3:**
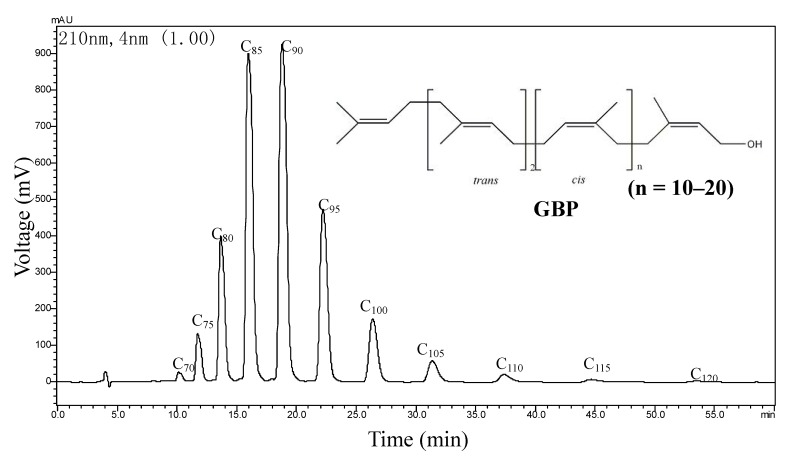
AD-HPLC-chromatogram (210 nm) and chemical structure of GBP.

**Figure 4:**
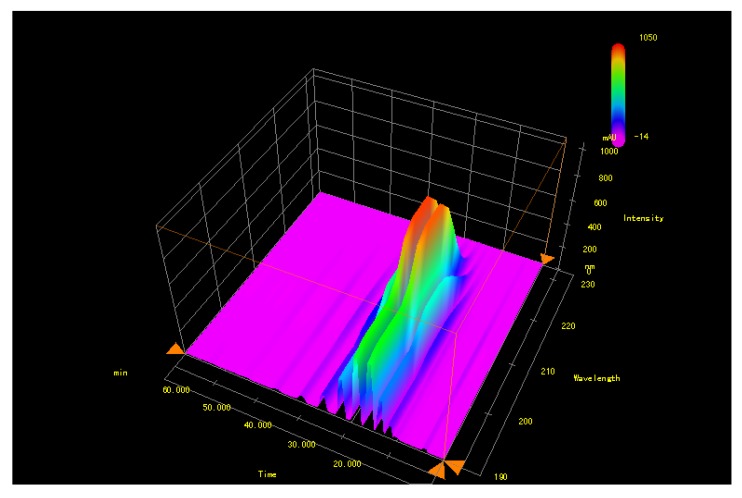
D-DAD-HPLC-chromatogram (190–232 nm) of GBP.

GBP (contents over 98%) separated from GBL lipids were determined by HPLC using an external standard method [21] and all the retention times and the absorption wavelengths of GBP match with those of standard polyprenols (C_70_, C_75_–C_105_, C_110_, C_115_, C_120_, Figure 3, Figure 4).

The present investigation supports that ethnobotanical uses of GBL relying on terpene trilactones and flavonoid glycosides, the active components of GBL might be responsible for their antibacterial activity [22]. Comparing with research on other different classes of compounds occurring in GBL extracts, the non-saponifiable lipids components were reported infrequently [1,23]. The separated and identified known compounds isophytol (**1**), nerolidol (**2**) and linalool (**3**) were reported for the first time among the components separated from GBL. Meanwhile, the 3D-DAD-HPLC-chromatogram (190–232 nm) of GBP was established for the first time and the maximum absorption wavelengths corresponding to each polyprenol homolog were recorded from the chromatogram for the first tome too.

### 2.2. Antibacterial and Antifungal Activity of GBL Lipids

Antibacterial and antifungal activity of the total non-saponifiable lipids (**S1**), the frozen sediment (**S2**), the light distillates (**S3**), the heavy distillates (**S4**) isolated by refrigeration and molecular distillation, the eight individual compounds and GBP was assessed in the present study at 500 μg/mL against three animal and plant pathogenic strains (*Salmonella enterica*, *Staphylocococus aureus* and *Aspergillus niger*) and their potencies were quantitatively assessed by their inhibition halos (Table 1), Minimum Inhibitory Concentration (MIC), Minimum Bactericidal Concentration (MBC) and Minimum Fungicidal Concentration (MFC) values (Table 2). Analysis of variance (Tukey’s test at 5% probability) indicated statistical differences (*p* < 0.05) among all the samples. The results from the diameters of inhibition halos indicated that nerolidol (**2**) showed the highest activity among all the tested samples and inhibited the growth of all the strains. MIC, MBC and MFC values for the examined strains sensitive (inhibition halos 16.2–20.1 mm) to nerolidol (**2**) were in the range of 3.9–15.6 μg/mL, 31.3–62.5 μg/mL and 62.5 μg/mL, respectively. GBP (inhibition halos 13.4–13.8 mm) showed the MIC values were 31.3 μg/mL, MBC and MFC were 125 μg/mL. The results from the inhibition halo diameters showed the activity could be ranked from high to low (the same below) in the following order: nerolidol (**2**), linalool (**3**), the heavy distillates (**S4**), the total non-saponifiable lipids (**S1**), the light distillates (**S3**), GBP and isophytol (**1**). These seven samples were effective in inhibiting the growth of all three examined strains. The five samples of *β*-sitosterol (**5**), the frozen sediment (**S2**), *β*-sitosterol acetate (**4**), ergosterol (**7**) and stigmasterol (**6**) were effective at inhibiting two of the types of bacteria examined in this study. *β*-Sitosterol-3-*O*-*β*-D-glucopyranoside (**8**) was only effective at inhibiting *Staphylocococus aureus*.

**Table 1:** omparison of the inhibition halos among different samples (Tukey’s test at 5% probability).

Samples ^▲^	Diameter of the inhibition halos (mm) ± SEM, n = 3					
	*S. enterica*		*S. aureus*		*A. niger*	
S1	15.1 ± 0.1 a		16.9 ± 0.1 a		14.4 ± 0.1 a	
S2	12.3 ± 0.1 b		12.8 ± 0.1 b		0.0	
S3	14.8 ± 0.1 a		14.8 ± 0.1 c		12.9 ± 0.1 b	
S4	16.0 ± 0.1 c		16.8 ± 0.1 a		15.0 ± 0.1 c	
GBP	13.5 ± 0.1 d		13.8 ± 0.1 d		13.4 ± 0.1 d	
C1	9.9 ± 0.1 e		13.4 ± 0.1 e		10.3 ± 0.1 e	
C2	17.4 ± 0.1 f		20.1 ± 0.1 f		16.2 ± 0.1 f	
C3	16.1 ± 0.1 c		14.9 ± 0.1 c		17.9 ± 0.1 g	
C4	10.9 ± 0.1 g		11.7 ± 0.1 g		0.0	
C5	12.1 ± 0.1 b		14.1 ± 0.1 d		0.0	
C6	7.7 ± 0.1 h		9.2 ± 0.1 h		0.0	
C7	8.1 ± 0.1 h		10.0 ± 0.1 i		0.0	
C8	11.1 ± 0.1 g		12.7 ± 0.1 b		0.0	
C1:GBP (1:1, wt/wt)	16.3 ± 0.1 *c	11.7 ^Δ^	17.1 ± 0.1 *a	13.6 ^Δ^	14.1 ± 0.1 *a	11.8 ^Δ^
C2:GBP (1:1, wt/wt)	16.2 ± 0.1 *c	15.4 ^Δ^	18.3 ± 0.1 *j	17.0 ^Δ^	15.3 ± 0.1 c	14.8 ^Δ^
C3:GBP (1:1, wt/wt)	15.1 ± 0.1 a	14.8 ^Δ^	15.2 ± 0.1 *c	14.4 ^Δ^	15.0 ± 0.1 c	15.6 ^Δ^
C4:GBP (1:1, wt/wt)	12.9 ± 0.1 i	12.2 ^Δ^	13.1 ± 0.1 b	12.8 ^Δ^	7.2 ± 0.1 h	6.7 ^Δ^
C5:GBP (1:1, wt/wt)	14.8 ± 0.1 *a	12.8 ^Δ^	16.3 ± 0.1 *k	14.0 ^Δ^	10.4 ± 0.1 *e	6.7 ^Δ^
C6:GBP (1:1, wt/wt)	10.2 ± 0.1 e	10.6 ^Δ^	10.2 ± 0.1 i	11.5 ^Δ^	8.3 ± 0.1 *i	6.7 ^Δ^
C7:GBP (1:1, wt/wt)	10.1 ± 0.1 e	10.8 ^Δ^	10.2 ± 0.1 i	11.9 ^Δ^	7.0 ± 0.1 h	6.7 ^Δ^
C8:GBP (1:1, wt/wt)	9.0 ± 0.1 j	12.3 ^Δ^	9.1 ± 0.1 h	13.2 ^Δ^	6.1 ± 0.1 j	6.7 ^Δ^
MN	0.0		20.4 ± 0.1		20.9 ± 0.4	
GS	20.2 ± 0.3		20.1 ± 0.5		0.0	

**S1**: Total non-saponifiable lipids; **S2**: Frozen sediment; **S3**: Light distillates; **S4**: Heavy distillates; GBP: *Ginkgo biloba* polyprenols; C1~C8: The separated compounds **1**~**8** reported in this paper; MN: Miconazole Nitrate (Positive control 1); GS: Gentamycin Sulfate (Positive control 2). ^Δ^ The theoretical values (C1~C8: GBP/1:1 arithmetic mean values) of the inhibition halos. * Having statistical difference compared with the corresponding theoretical values (Tukey’s HSD test, *p* < 0.05). The same lowercase letters in the same column indicate no statistical difference (Tukey’s HSD test, *p* > 0.05). ^▲^ All samples were examined at 500 μg/mL, and the mixture groups’ total mass concentration was 500 μg/mL.

**Table 2:** inimum inhibitory/bactericidal/fungicidal concentration (*μ*g/mL) of different samples.

Samples	MIC, MBC and MFC values (*μ*g/mL)					
	*S. enterica*		*S. aureus*		*A. niger*	
S1	15.6	62.5 *	15.6	62.5 *	31.3	125 **
S2	62.5	125 *	62.5	125 *	/	/
S3	31.3	125 *	31.3	125 *	31.3	125 **
S4	31.3	62.5 *	15.6	62.5 *	31.3	62.5 **
GBP	31.3	125 *	31.3	125 *	31.3	125 **
C1	31.3	125 *	31.3	62.5 *	31.3	125 **
C2	15.6	62.5 *	3.9	31.3 *	15.6	62.5 **
C3	15.6	62.5 *	31.3	62.5 *	7.8	31.3 **
C4	62.5	250 *	62.5	250 *	/	/
C5	31.3	125 *	31.3	125 *	/	/
C6	125	>250 *	125	>250 *	/	/
C7	125	>250 *	62.5	250 *	/	/
C8	62.5	250 *	62.5	125 *	/	/
C1:GBP (1:1, wt/wt)	7.8 ^Δ^		15.6 ^Δ^		15.6 ^Δ^	
C2:GBP (1:1, wt/wt)	15.6 ^Δ^		3.9 ^Δ^		15.6 ^Δ^	
C3:GBP (1:1, wt/wt)	15.6 ^Δ^		31.3 ^Δ^		15.6 ^Δ^	
C4:GBP (1:1, wt/wt)	31.3 ^Δ^		31.3 ^Δ^		62.5 ^Δ^	
C5:GBP (1:1, wt/wt)	15.6 ^Δ^		15.6 ^Δ^		31.3 ^Δ^	
C6:GBP (1:1, wt/wt)	62.5 ^Δ^		62.5 ^Δ^		62.5 ^Δ^	
C7:GBP (1:1, wt/wt)	62.5 ^Δ^		62.5 ^Δ^		62.5 ^Δ^	
C8:GBP (1:1, wt/wt)	62.5 ^Δ^		62.5 ^Δ^		62.5 ^Δ^	

* MBC values; ** MFC values. **S1**: Total non-saponifiable lipids; **S2**: Frozen sediment; **S3**: Light distillates; **S4:** Heavy distillates; GBP: *Ginkgo biloba* polyprenols; C1–C8: The separated compounds **1**~**8** reported in this paper. ^Δ^ The mixture groups’ total mass concentration.

All the samples separated from the light distillates (**S3**) and the heavy distillates (**S4**) were effective at inhibiting the growth of the three examined strains. None of the samples separated from the frozen sediment (**S2**) were active against *Aspergillus niger*.

Nerolidol showed the highest activity among all the tested samples and inhibited the growth of all the strains used in this study. It was suggested that nerolidol possesses antifungal activity against *T. mentagrophytes* and the activity may lead to irreversible cellular disruption [24]. Nerolidol enhanced the susceptibility of *Staphylococcus aureus* to ciprofloxacin, clindamycin, erythromycin, gentamicin, tetracycline, and vancomycin. Nerolidol also sensitized *Escherichia coli* to polymyxin B [25]. Consequently, it would be meaningful to research on expanding the antimicrobial spectrum of nerolidol in the future. In our previous study we reported on hepatoprotective and antitumour effects of the polyprenols of GBL in rats [7,26,27]. This study supports further research as there are no previous records on the anti-microorganism activity of GBP. Especially, the polyprenols are an important component in the non-saponifiable lipids (**S1**, GBP content > 40%) and in the heavy distillates (**S4**, GBP content > 80%) of GBL [9,28]. Besides, there is new interest in finding out the other lipids in the heavy distillates **S4** having higher antibacterial and antifungal activities than the polyprenols from the above results. Therefore, it can be inferred GBP have synergistic inhibitory effects on microorganisms with other lipids, so it was indispensable to research the synergistic inhibitory effect on GBP with other lipids compounds in the next step.

### 2.3. Synergistic Antibacterial and Antifungal Effects on GBP with Separated Compounds

The method for assessing synergistic antibacterial/antifungal effects of GBP with separated compounds was by comparing the diameters of inhibition halos between theoretical values and actual values of mixture groups (compounds **1**–**8**:GBP, 1:1, wt/wt) by analysis of variance (Tukey’s test at 5% probability) from Table 1, and the Fractional Inhibitory Concentration (FIC) indexes from Table 3. The results showed that two mixture groups C1:GBP and C5: GBP had statistically significant differences (Tukey’s test, *p* > 0.05) compared with the corresponding theoretical values of diameters of inhibition halos among all the mixture groups against all the tested strains. The mixture group C1:GBP had the most significant statistical difference (Tukey’s test, F = 690.036, *p* = 0.001) against *Salmonella enterica* compared with other mixture groups. The FIC index of C1:GBP against *Salmonella enterica* was 0.25, that was lowest in all mixture groups against all the tested strains. This result suggested that the GBP with isophytol (**1**) mixture group had the strongest synergistic effect against *Salmonella enterica* of all mixture groups against the three tested strains. Isophytol is an important component of many essential oils with reported antimicrobial activity [29,30,31]. No studies on synergistic antimicrobial effects of isophytol and GBP have been reported. From here we saw that it was necessary to establish the optimal proportions for the synergistic effect of GBP with isophytol against *Salmonella enterica* in the next study.

**Table 3:** ractional inhibitory concentration (FIC) index used to determine the type of interactions.

Samples	FIC index		
	*S. enterica*	*S. aureus*	*A. niger*
C1:GBP (1:1, wt/wt)	0.25 *	0.5 *	0.5 *
C2:GBP (1:1, wt/wt)	0.75 ^Δ^	0.56 ^Δ^	0.75 ^Δ^
C3:GBP (1:1, wt/wt)	0.75 ^Δ^	1 ^Δ^	1.25 ^▲^
C4:GBP (1:1, wt/wt)	0.75 ^Δ^	0.75 ^Δ^	1 ^Δ^
C5:GBP (1:1, wt/wt)	0.5 *	0.5 *	0.5 *
C6:GBP (1:1, wt/wt)	1.25 ^▲^	1.25 ^▲^	1^Δ^
C7:GBP (1:1, wt/wt)	1.25 ^▲^	1.5 ^▲^	1^Δ^
C8:GBP (1:1, wt/wt)	1.5 ^▲^	1.5 ^▲^	1^Δ^

C1–C8: The separated compounds 1–8 reported in this paper. * Synergistic effect (0 < FIC index ≤ 0.5); ^△^Additive effect (0.5 < FIC index ≤ 1) and ^▲^Indifferent effect (1 < FIC index ≤ 4).

### 2.4. Optimal Proportioning Design of Synergistic Effect on GBP with Isophytol against Salmonella Enterica

**Figure 5:**
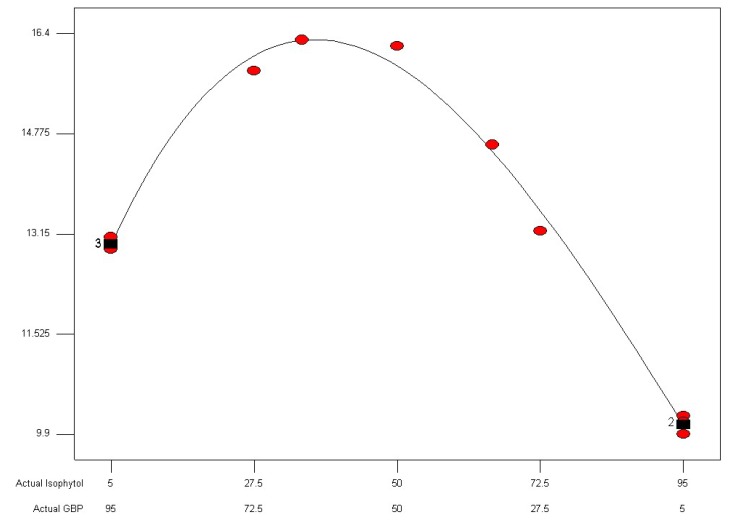
Diameters of inhibition halos of isophytol and GBP mixture in different proportions against *Salmonella enterica* drawn by cubic curve fitting.

**Figure 6:**
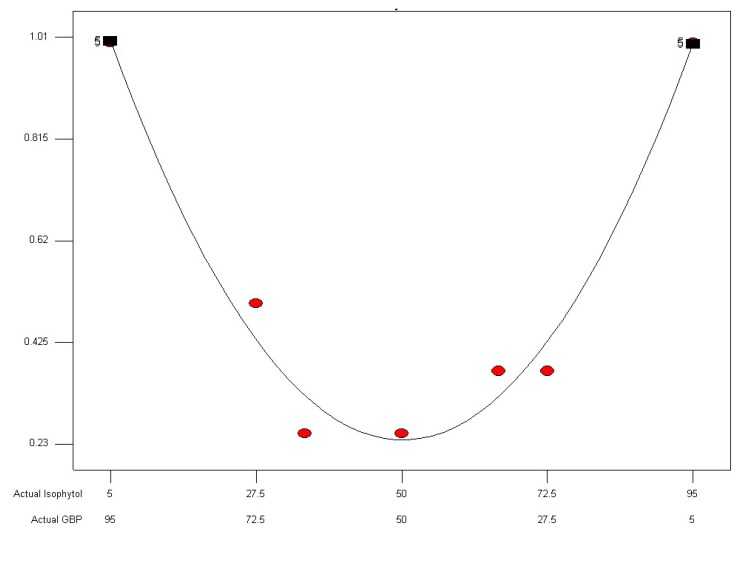
FIC index of isophytol and GBP mixture in different proportions against *Salmonella enterica* drawn by cubic curve fitting.

The optimal proportion determination of synergistic effect on GBP with isophytol against *Salmonella enterica* was based on the Mixture Design (D-optimal, two mixture components, two factors) method (Table 4) by ANOVA analysis with a cubic model. The final equations in terms of actual components was:

R_1_= 0.011307 × A + 0.012271 × B − 3.76438 × 10^−4^ × A × B + 1.01315×10^−6^ × A × B × (A−B) (1)

where R_1_ = FIC index, A = isophytol, B = GBP, F = 6320.36, *p* < 0.0001; R_2_ = 0.092397 × A + 0.11760 × B − 2.14996 × 10^−3^×A×B + 7.69784 × 10^−6^ × A × B × (A−B) (R_2_ = diameters of inhibition halos, A = isophytol, B = GBP, F = 552.29, *p* < 0.0001). The result showed the lowest FIC index response value was 0.2452 (Figure 5) and the highest response value of diameters of inhibition halos was 16.3019 mm (Figure 6). Under these conditions, the theoretical proportion of isophytol and GBP was 38.19%:61.81% (wt/wt) that was determined as the optimal proportion for synergistic effect on GBP with isophytol against *Salmonella enterica*. The actual experimental values of FIC index and diameters of inhibition halos at the 38.19%:61.81% (wt/wt) proportion of isophytol:GBP against *Salmonella enterica* were 0.25 and 16.5 mm, respectively. The relative error between the actual experimental values and the theoretical values using this design and analysis method was 1.96% (FIC index) and 1.22% (diameters of inhibition halos), respectively. It can thus be inferred that this design and analysis method was both reasonable and reliable.

**Table 4:** ptimal proportioning design * of synergistic effect on GBP with isophytol against *Salmonella enterica.*

Std. ^Δ^	Run	ComponentA: Isophytol (%) ^▲^	ComponentB: GBP (%) ^▲^	Response1: FIC index	Response2: Diameter of the inhibition halos (mm)
10	1	95.00	5.00	1	10.1
9	2	95.00	5.00	1	9.9
4	3	27.50	72.50	0.37	15.8
13	4	5.00	95.00	1	13.1
8	5	5.00	95.00	1	13.0
5	6	72.50	27.50	0.5	13.2
12	7	95.00	5.00	1	10.1
3	8	50.00	50.00	0.25	16.2
2	9	95.00	5.00	1	10.2
7	10	65.00	35.00	0.37	14.6
11	11	5.00	95.00	1	12.9
1	12	5.00	95.00	1	13.0
6	13	35.00	65.00	0.25	16.3

* It was based on the Mixture Design (D-optimal, two mixture components, two factors) method in Design Expert 7.1.3 Software and all samples were examined at 500 *μ*g/mL (total mass concentration). ^Δ^ Random generation. ^▲^ The limits: 5%–95%.

## 3. Experimental

### 3.1. Materials

The dried GBL were collected in October 2011 from China’s Jiangsu Province. This plant was identified and authenticated by Prof. Cheng-Zhang Wang at the Institute of Chemical Industry of Forestry Products, CAF in China. Three types of strains (*Salmonella enterica* ATCC 14028; *Staphylocococus aureus* ATCC 25923; *Aspergillus niger* ATCC 16404) bought from the China Center for Type Culture Collection (CCTCC, Wuhan, China) were used. A Bruker AV-300 NMR instrument was used for compound identification, using CDCl_3_ or DMSO-d_6_ as solvents and TMS as internal standard. HPTLC plates (silica gel 60) were obtained from Merck. The standard polyprenols (C_70_, C_75_–C_105_, C_110_, C_115_, C_120_) were purchased from Larodan Fine Chemical Co., Ltd, (Malmö, Sweden). HPLC measurements was performed at room temperature with a Shimadzu SPD-20A instrument equipped with DAD detector (210 nm) and a 2.5 *μ*m Thermo BDS HYPERSIL C18 (150 × 4.6 mm) column, using 64/36 isopropanol/methanol solvent mixture as the eluent at 0.5 mL/min for 60 min.

### 3.2. Extraction and Isolation

Shade air-dried (at room temperature) and pulverized (over 80 mesh sieve) GBL (10 kg) were extracted three times with 30 L (total) petroleum ether (b.p. 60–90 °C) for 24 h at 65–70 °C and concentrated to give an extract (500 g) which was mixed with 5% NaOH-EtOH solution (6 L) for 3 h at room temperature. The hydrolysate was extracted with petroleum ether (6 L) three times. The collected organic phases were washed with water to neutrality and dried with anhydrous Na_2_SO_4_. The solvent was evaporated under vacuum to give the total non-saponifiable lipid extract (**S1**, 350 g) which was dissolved in a solvent mixture (acetone : methanol = 85:15, v/v) for a solid-liquid ratio of 1:6–1:8 (g/mL), then refrigerated for 2 h at −15 °C [7,9,28]. As a result, the frozen sediment (**S2**, 35 g) was obtained by quickly filtering at low temperature the solids from the refrigerated solution and the dissolved matter was concentrated to yield a product as a brown oil (215 g). The brown oil was fractionated by molecular distillation at a feed temperature of 60 °C, distillation temperature of 280 °C, feed flow rate of 180 mL/h, scraper rate of 300 rpm, and operating pressure of 0.1-0.5 Pa to give the light distillates (**S3**) as a yellow oil (44 g) and the heavy distillates (**S4**) as a dark brown oil (168 g).

The light distillates were subjected to silica gel column chromatography (Merck, Kieselgel 60; 0.063–0.2 mm particle size; 5 × 100 cm). The fractions were eluted step by step with petroleum/ethyl ether (100%:0–90%:10%, v/v, 10 × 250 mL). Fractions of similar composition as determined by TLC analysis were pooled. Compound **1** (178 mg) was obtained from the petroleum/ethyl ether (95%: 5%, v/v) portion that was separated on a HPTLC plate developed with petroleum/ethyl ether (91%: 9%, v/v). Iodine was chosen for color development. The petroleum/ethyl ether (92%: 8%, v/v) portion was subjected to gel permeation column chromatography (GE, Sephadex LH-20; 1.5 × 100 cm) and eluted with CHCl_3_/MeOH (60%: 40%, v/v, 10 × 15 mL) in order to purify compound **2** (51 mg). Compound **3** (20 mg) was obtained from the petroleum/ethyl ether (90%: 10%, v/v) portion that was separated by HPTLC developed by petroleum/ethyl ether (85%:15%,v/v). 5% Anisaldehyde sulphuric acid–EtOH solution was chosen for color development.

GBP were further purified from a portion (10 g) of the heavy distillates **S4** by flash column chromatography (Merck, Kieselgel 60; 0.063–0.2 mm particle size; 6 × 80 cm), using petroleum ether (5 × 250 mL) and 1%, 2%, 3% ethyl ether/petroleum ether (5 × 250 mL) as eluents [7,9,28]. The polyprenols (2.5 g) were obtained from the petroleum/ethyl ether (97%:3%, v/v) portion.

The frozen sediment (**S2**, 32 g) was dissolved in CHCl_3_ (30 mL) and the CHCl_3_solution was subjected to silica gel column chromatography (Merck, Kieselgel 60; 0.063~0.2 mm particle size; 5 × 80 cm). The fractions were eluted step by step with CHCl_3_/MeOH (100%:0~85%:15%, v/v, 10 × 250 mL). Fractions of similar composition as determined by TLC analysis were pooled. The 100% CHCl_3_ portion was recrystallized from 2-propanol to yield compound **4** (290 mg). The CHCl_3_/MeOH (99%:1%, v/v) portion was subjected to gel permeation column chromatography (GE, Sephadex LH-20; 1.5 × 100 cm) and eluted with CHCl_3_/MeOH (50%: 50%, v/v, 10 × 10 mL) to isolate in order compounds **5** (29 g, cyclohexanone), **6** (116 mg, *n*-pentanol) and **7** (105 mg, Et_2_O). The CHCl_3_/MeOH (90%:10%, v/v) portion was recrystallized from chloroform at low temperature to yield compound **8** (203 mg). ^1^H and ^13^C-NMR data of the structures of the eight compounds were as follows:

*Isophytol* (**1**): ^1^H-NMR (CDC1_3_) δ (ppm): 5.87–5.96 (1H, dd, H-2), 5.17–5.23 (1H, dd, H-1a), 5.01–5.05 (1H, dd, H-1b), 1.54–1.56 (2H, m, H-4), 1.45–1.53 (1H, m, H-15), 1.32–1.43 (2H, m, H-7, 11), 1.27 (3H, br-s, H-20); The position of this signal varied from 1.03–1.30 ppm for the other saturated methylene protons (total 16H); 0.88 (6H, s, H-16,17), 0.86 (3H, s, H-19), 0.83 (3H, s, H-18). ^13^C-NMR (CDC1_3_) δ (ppm): 145.3 (C-2), 111.4 (C-1), 73.3 (C-3), 42.7 (C-4), 39.4 (C-14), 37.5 (C-12), 37.4 (C-10), 37.4 (C-8), 37.3 (C-6), 32.8 (C-7), 28.0 (C-15), 27.7 (C-20), 24.8 (C-13), 24.4 (C-9), 22.7 (C-16), 22.6 (C-17), 21.4 (C-5), 19.7 (C-18), 19.6 (C-19).

*Nerolidol* (**2**): ^1^H-NMR (CDC1_3_) δ (ppm): 5.91–5.96 (1H, dd, H-2), 5.18–5.24 (1H, dd, H-1a), 5.03–5.07 (1H, dd, H-1b), 5.10–5.14 (1H, m, H-6), 5.06–5.09 (1H, m, H-10), 1.96–2.10 (6H, m, H-5,8,9), 1.68 (3H, s, H-12), 1.60 (6H, s, H-13,14), 1.51–1.58 (2H, m, H-4), 1.27 (3H, br-s, H-15). ^13^C-NMR (CDC1_3_) δ (ppm): 145.0 (C-2), 135.5 (C-7), 131.3 (C-11), 125.0 (C-6), 124.2 (C-10), 111.6 (C-1), 73.4 (C-3), 42.0 (C-4), 39.6 (C-8), 27.8 (C-15), 26.6 (C-9), 25.6 (C-12), 22.6 (C-5), 17.6 (C-13), 15.9 (C-14).

*Linalool* (**3**): ^1^H-NMR (CDC1_3_) δ (ppm): 5.86–5.96 (1H, dd, H-2), 5.18–5.24 (1H, dd, H-1a), 5.10–5.14 (1H, m, H-6), 5.04–5.08 (1H, dd, H-1b), 1.98–2.07 (2H, m, H-5), 1.68 (3H, s, H-8), 1.60 (3H, s, H-9), 1.56–1.59 (1H, m, H-4a), 1.53–1.55 (1H, m, H-4b), 1.28 (3H, br-s, H-10). ^13^C-NMR (CDC1_3_) δ (ppm): 145.0 (C-2), 131.8 (C-7), 124.3 (C-6), 111.6 (C-1), 73.4 (C-3), 42.0 (C-4), 27.8 (C-10), 25.6 (C-8), 22.8 (C-5), 17.6 (C-9).

*β-Sitosterol acetate* (**4**): ^1^H-NMR (CDC1_3_) δ (ppm): 5.37, 5.38 (1H, br-d, *J* = 4.2Hz, 6-H), 4.55–4.66 (1H, m, 3-H), 2.03 (3H, s, CH_3_COO-); The position of other signals overlapped with the corresponding protons of compound **5**. The signals of the alkali-hydrolysis product were same as the signals of the corresponding protons of compound **5**.

*β-Sitosterol* (**5**): ^1^H-NMR (CDC1_3_) δ (ppm): 5.34, 5.36 (1H, br-d, *J* = 4.9Hz, 6-H), 3.48–3.58 (1H, m, 3-H); The position of this signal varied from 1.05–2.33 ppm for the other saturated methylene and methine protons (total 30H); 1.01 (3H, s, 19-H), 0.91, 0.93 (3H, d, *J*=6.5 Hz, 26-H), 0.85, 0.87 (3H, d, 21-H), 0.80–0.85 (3H, t, 29-H), 0.80, 0.82 (3H, d, 27-H), 0.68 (3H, s, 18-H). ^13^C-NMR (DMSO-d_6_) δ (ppm): 140.7 (C-5), 121.7 (C-6), 71.8 (C-3), 56.2 (C-14), 56.0 (C-17), 50.1 (C-9), 45.8 (C-24), 42.2 (C-4), 39.7 (C-12), 37.2 (C-1), 36.5 (C-10), 36.1 (C-20), 33.9 (C-7), 31.9 (C-8,22), 31.6 (C-2), 29.1 (C-23), 28.2 (C-16), 26.0 (C-28), 24.3 (C-15), 23.0 (C-26,27), 21.0 (C-11), 19.8 (C-19), 19.0 (C-13), 11.8 (C-18), 18.8 (C-21, 25), 11.9 (C-29).

*Stigmasterol* (**6**): ^1^H-NMR (CDC1_3_) δ (ppm): 5.34, 5.36 (1H, d, *J* = 5.1 Hz, H-6), 5.12–5.20 (1H, dd, *J* = 8.4 Hz, H-22), 4.98–5.06 (1H, dd, *J* = 8.4 Hz, H-23), 3.47–3.57 (1H, m, H-3). The position of this signal varied from 1.04–2.33 ppm in the other saturated methylene and methine protons (total 26H); 1.01, 1.03 (3H, d, H-21), 1.01 (3H, br-s, H-19), 0.84, 0.86 (3H, d, H-26), 0.81, 0.83 (3H, d, H-27), 0.79–0.84 (3H, t, H-29), 0.70 (3H, br-s, H-18). ^13^C-NMR (CDC1_3_) δ (ppm): 37.3 (C-1), 31.7 (C-2), 71.8 (C-3), 42.2 (C-4), 140.8 (C-5), 121.7 (C-6), 31.9 (C-7), 31.9 (C-8), 50.2 (C-9), 36.5 (C-10), 21.1 (C-11), 39.7 (C-12), 42.3 (C-13), 56.9 (C-14), 24.4 (C-15), 28.9 (C-16), 56.0 (C-17), 12.0 (C-18), 19.4 (C-19), 40.5 (C-20), 19.0 (C-21), 138.3 (C-22), 129.3 (C-23), 51.2 (C-24), 31.7 (C-25), 21.1 (C-26), 21.2 (C-27), 25.4 (C-28), 12.2 (C-29).

*Ergosterol* (**7**): ^1^H-NMR (CDC1_3_) δ (ppm): 5.56–5.58 (1H, dd, H-6), 5.37–5.39 (1H, ddd, H-8), 5.20–5.27 (1H, dd, H-23), 5.13–5.20 (1H, dd, H-22), 3.58–3.75 (1H, m, H-3). The position of this signal varied from 1.20–2.50 ppm in the other saturated methylene and methine protons (total 21H); 1.03, 1.05 (3H, d, *J* = 6.6 Hz, H-21), 0.95 (3H, s, H-19), 0.91, 0.93 (3H, d, *J* = 6.8 Hz, H-28), 0.83, 0.85 (3H, d, *J* = 6.7 Hz, H-26), 0.81, 0.84 (3H, d, *J* = 6.7 Hz, H-27), 0.63 (3H, S, H-18). ^13^C-NMR (CDC1_3_) δ (ppm): 141.3 (C-8), 139.8 (C-5), 135.6 (C-22), 132.0 (C-23), 119.6 (C-6), 116.3 (C-7), 70.4 (C-3), 55.8 (C-17), 54.6 (C-14), 46.3 (C-9), 42.8 (C-13), 42.8 (C-24), 40.8 (C-4), 40.4 (C-20), 39.1 (C-12), 37.0 (C-10), 38.4 (C-1), 33.1 (C-25), 32.0 (C-2), 28.3 (C-16), 23.0 (C-15), 21.1 (C-21), 21.1 (C-11), 19.9 (C-27), 19.6 (C-26), 17.6 (C-19), 16.3 (C-28), 12.0 (C-18).

*β-Sitosterol-3-O-β-D-glucopyranoside* (**8**): ^1^H-NMR (CDC1_3_) δ (ppm): 5.34, 5.36 (1H, br-d, *J* = 4.9 Hz, H-6), 4.40, 4.43 (1H, d, *J* = 7.7 Hz, H-1′), 3.74–3.87 (2H, ddd, H-6′). The position of this signal varied from 3.30–3.50 ppm in the other protons (4H) attached to carbon connected to oxygen atoms in the glucose ring structure. The position of this signal varied from 1.06–2.44 ppm in the other saturated methylene and methine protons (total 29H); 1.00 (3H, s, 19-H), 0.91, 0.93 (3H, d, *J* = 6.4 Hz, H-27), 0.85, 0.87 (3H, d, 21-H), 0.80–0.85 (3H, t, 29-H), 0.80, 0.82 (3H, d, 27-H), 0.68 (3H, s, 18-H). ^13^C-NMR (DMSO-d_6_+CDCl_3_) δ (ppm): 140.4 (C-5), 121.9 (C-6), 101.2 (C-1′), 78.8 (C-3), 77.6 (C-3′), 75.7 (C-5′), 73.8 (C-2′), 71.2 (C-4′), 62.7 (C-6′), 56.7 (C-14), 56.0 (C-17), 50.2 (C-9), 45.8 (C-24), 42.3 (C-13), 39.7 (C-4), 38.9 (C-12), 37.3 (C-1), 36.7 (C-10), 36.1 (C-20), 33.9 (C-22), 31.9 (C-7), 29.7 (C-8), 29.6 (C-2), 29.2 (C-25), 28.1 (C-16), 26.1 (C-23), 24.2 (C-15), 23.0 (C-28), 21.0 (C-11), 19.7 (C-27), 19.3 (C-19), 19.0 (C-26), 18.7 (C-21), 11.9 (C-29), 11.8 (C-18).

### 3.3. HPLC Analysis

The concentration of the GBP sample was 4.38 mg/mL and the injection volume was 5 μL. The retention time of different carbon chain GBPs is 10.127 min (C_70_), 11.724 min (C_75_), 13.651 min (C_80_), 15.966 min (C_85_), 18.782 min (C_90_), 22.216 min (C_95_), 26.393 min (C_100_), 31.361 min (C_105_), 37.368 min (C_110_), 44.663 min (C_115_) and 53.519 min (C_120_), respectively. The absorption wavelength range of GBP is 190–232 nm in scanning 190–800 nm with DAD detector and the maximum absorption wavelengths of GBPs are 207 nm (C_70_), 207 nm (C_75_), 208 nm (C_80_), 210 nm (C_85_), 210 nm (C_90_), 208 nm (C_95_), 207 nm (C_100_), 207 nm (C_105_), 207 nm (C_110_), 207 nm (C_115_) and 206 nm (C_120_), respectively.

### 3.4. Determination of Antibacterial and Antifungal Activity

Antibacterial and antifungal tests of selected stains were carried out using a disc-diffusion method [32]. A small sterile cotton swab was dipped into the 24-h-old culture of stains and was inoculated by streaking the swab over the entire agar surface. After inoculation the plates were allowed to dry at room temperature in laminar chamber. The filter paper discs (6 mm) loaded with 100 μL of sample were placed on the surface of the agar plates. After 5 min the plates were incubated at 37 °C for 24 h. Miconazole nitrate (Sigma SM351201, 1 g) and gentamycin sulfate (Sigma G3632, 100 mg) were used as positive control and the respective solvent as negative control. After 24 h of incubation, the diameter was observed for inhibition halos (measured in mm including disc size). All tests were performed in triplicate and observed values of inhibition halos were expressed as mean value with standard error of means (SEM).

### 3.5. Determination of Minimum Inhibitory Concentration (MIC), Minimum Bactericidal Concentration (MBC), Minimum Fungicidal Concentration (MFC), FIC (Fractional Inhibitory Concentration) Index and Determination of the Type of Interactions of Antibacterial and Antifungal Activity

MIC, MBC and MFC were determined using the broth-dilution method. MIC was performed at seven concentrations of samples (250, 125, 62.5, 31.3, 15.6, 7.8, 3.9 *μ*g/mL) following serial dilution technique. All the wells showing no visible growth of strains were subcultured and incubated at 37 °C (*Salmonella enterica*, *Staphylocococus aureus*) and 28 °C (*Aspergillus niger*) overnight. The highest dilution showing 100% inhibition was recorded as MBC or MFC [12]. The FIC is the concentration that kills when used in combination with another agent divided by the concentration that has the same effect when used alone [33]. The FIC index for the combination of A and B is the sum of their individual FIC values. The determination of the type of interactions referred to synergistic effect (0 < FIC index ≤ 0.5), additive effect (0.5 < FIC index ≤ 1), indifferent effect (1 < FIC index ≤ 4) and antagonism effect (FIC index > 4) [34,35,36].

### 3.6. Optimal Proportioning Design of Synergistic Effect on GBP with Isophytol against Salmonella Enterica

This design was based on the Mixture Design (D-optimal, two mixture components, two factors, the limits: 5%–95%) option in the Design Expert 7.1.3 Software that generated the experimental scheme (13 standard/run) randomly. All samples were examined at 500 μg/mL (total mass concentration). The components A and B were isophytol and GBP, respectively. The responses 1 and 2 were the FIC index and diameters of inhibition halos, respectively.

## 4. Conclusions

The eight known compounds isophytol (**1**), nerolidol (**2**), linalool (**3**), *β*-sitosterol acetate (**4**), *β*-sitosterol (**5**), stigmasterol (**6**), ergosterol (**7**) and *β*-sitosterol-3-*O*-*β*-D-glucopyranoside (**8**) were separated from GBL by chromatography and identified by NMR. The separated and identified compounds **1**, **2** and **3** were reported for the first time from GBL. The 3D-DAD-HPLC-chromatogram (190–232 nm) of GBP was recorded for the first time. Meanwhile, this study provides the first evidence of the antibacterial/antifungal activities and synergistic effect on GBP with compounds separated from GBL lipids against *Salmonella enterica*, *Staphylocococus aureus* and *Aspergillus niger*. Nerolidol (**2**) showed the highest activity among all the tested samples, and the GBP with isophytol (**1**) mixture group had the strongest synergistic effect against *Salmonella enterica* among all mixture groups against the three tested strains. The proportion of isophytol and GBP of 38.19%:61.81% (wt/wt) was determined as the optimal proportion of synergistic effect on GBP with isophytol against *Salmonella enterica*. This study provides a new scientific basis for the ethnomedical use of GBL against bacterial and fungal diseases of animals and plants.
